# Supplementary material for: Learning curve and surgical outcome of robotic assisted colorectal surgery with ERAS program
Source: Sci Rep. 2022 Nov 29;12:20566. doi: 10.1038/s41598-022-24665-w (PMC9709162; doi:10.1038/s41598-022-24665-w)
Supplement: Supplementary file 1 — Supplementary Information 1. [file 41598_2022_24665_MOESM1_ESM.zip › LSC Raw data-Ñ[▒K/103/O_ñw╝╨░O▒Kñσ.pdf]

正式摘要(電子簽章：000000203050816)

請選擇...reporter

臺中榮民總醫院

[redacted]

- 手術日期：2020/11/12
- 手術醫師：VS 蔣鋒帆，CR 楊曙榮，CR 黃偘霖，R 陳璿夫，R 陳冠樺
- Anesthesia：GE
- Position：Lithotomy
- Aseptic agents：Soap，Betadine Alcohol，Betadine Aqua

Diagnosis

- Pre-operative diagnosis : Rectum tumor for lapa OP
- Post-operative diagnosis : Ditto.

Surgical Indication

- Tumor resection

Operation

- 1. Laparoscopic low anterior resection, end-to-end anastomosis, EndoGIA 60/.38mm, CEEA- 29mm. Bilateral doughnut: intact, air tight test: done, no air leak.
  2. High ligation (+) above the left colic artery.
  3. Cut margin: proximal 15 cm, distal 3 cm.
  5. Take down of splenic flexure: (-).
  6. No drain placed.

Finding

- 1. A 4x6 cm ulcerative lesion at upper rectum.
  2. There were no liver metastases or peritoneal seeding.
  3. There were no synchronous polyps or tumor at resected specimen.
  4. There were some clear ascites at pelvics.
  5. Surgiwrap +
  6. No drain tube

Procedure

- General endotracheal anesthesia was induced and then the patient was placed in modified Trendelenburg lithotomy position. Preoperative antibiotics were given. A Foley catheter and nasogastric tube were placed. The abdomen was prepped and draped in standard sterile fashion. A small vertical incision was made at midline and 12mm trochars were inserted into the peritoneal cavity by open method. Peumoperitoneum was established with CO2 inflation. Laparoscopy was inserted and then another three 5mm trochars were inserted over LLQ ,LUQ and RUQ of abdomen.One 12mm trochars were inserted over RLQ. The operative findings were mentioned as above. The sigmoid colon was held by grasper and the peritoneum overlying the mesentery was scored with electrocautery. The inferior mesentery artery was identified, ligated below the left colic artery with endoclips, and transected. Dissection was done above the Gerota's fascia and toward the peritoneal attachments. The colon was mobilized from retroperitoneum overlying the sacral promontory and into the pelvis. The incision was extended laterally and medially across the peritoneal reflection. The mesorectum was then mobilized. Distal descending colon and rectum were mobilized. The mesorectum was then mobilized using sharp dissection until the levator ani muscle was identified.The rectum was divided with EndoGIA 60/3.8. The midline wound was enlarged and the rectum was pulled out. The distal descending colon was transected and the specimen was removed from the operative field. The cap of CEEA was applied at the distal end of the descending colon. Pneumoperitoneum was constructed again. End-to-end anastomosis was established with

- CDH 29mm. Surgiwrap was placed at midline wound. The fascia was closed with interrupted sutures of No.1 Vicryl.The skin was approximated with 3-0 Vicryl and Surgiseal. The patient tolerated the procedure well and was brought to the POR in stable condition
- Estimated blood loss: minimum
- 器械數量: 正確
- 紗布數量: 正確

Image

- PAINT
  - abdomen

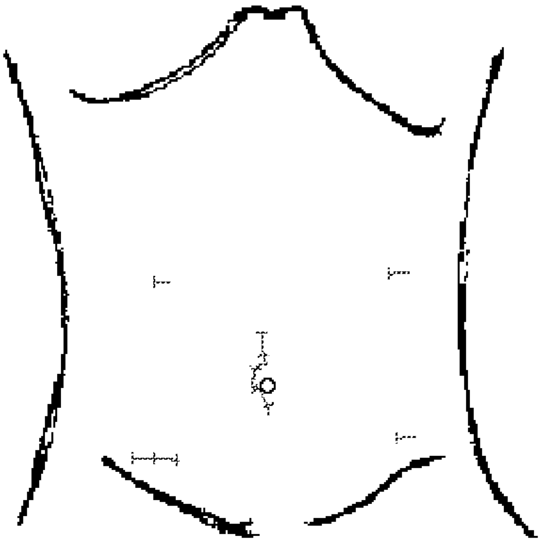

  - colon & rectum

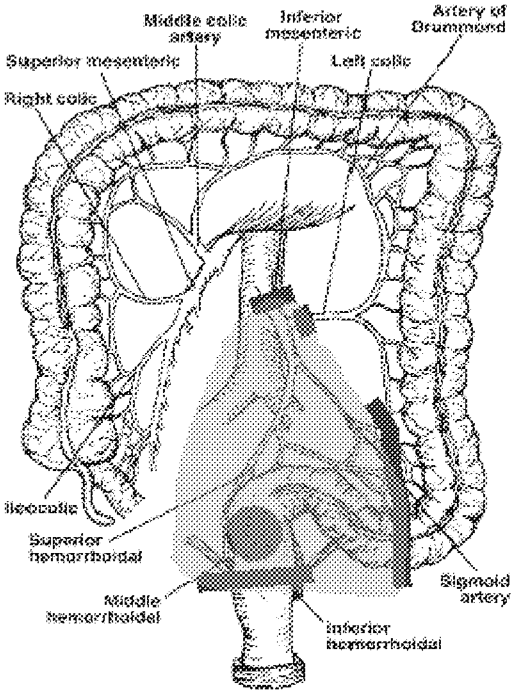

主任 2062F蔣鋒帆 / 醫師 2292G楊曙榮

正式摘要**(**電子簽章：**000000203050816)**

請選擇...reporter

doctorRank:B
